# Decision Tree With Only Two Musculoskeletal Sites to Diagnose Polymyalgia Rheumatica Using [^18^F]FDG PET-CT

**DOI:** 10.3389/fmed.2021.646974

**Published:** 2021-02-17

**Authors:** Anthime Flaus, Julie Amat, Nathalie Prevot, Louis Olagne, Lucie Descamps, Clément Bouvet, Bertrand Barres, Clémence Valla, Sylvain Mathieu, Marc Andre, Martin Soubrier, Charles Merlin, Antony Kelly, Marion Chanchou, Florent Cachin

**Affiliations:** ^1^Department of Nuclear Medicine, Saint-Etienne University Hospital, University of Saint-Etienne, Saint-Etienne, France; ^2^Department of Nuclear Medicine, Jean Perrin Oncology Institute of Clermont-Ferrand, Clermont-Ferrand, France; ^3^Institut national de la santé et de la recherche médicale, U 1059 Sainbiose, Université Jean Monnet, Saint-Etienne, France; ^4^Department of Internal Medicine, Gabriel Montpied University Hospital, University of Clermont-Ferrand, Clermont-Ferrand, France; ^5^Department of Rheumatology, Gabriel Montpied University Hospital, University of Clermont-Ferrand, Clermont-Ferrand, France

**Keywords:** polymyalgia rheumatica, inflammatory rheumatism, [18F]FDG PET-CT, decision-tree algorithm, machine learning

## Abstract

**Introduction:** The aim of this study was to find the best ordered combination of two FDG positive musculoskeletal sites with a machine learning algorithm to diagnose polymyalgia rheumatica (PMR) vs. other rheumatisms in a cohort of patients with inflammatory rheumatisms.

**Methods:** This retrospective study included 140 patients who underwent [^18^F]FDG PET-CT and whose final diagnosis was inflammatory rheumatism. The cohort was randomized, stratified on the final diagnosis into a training and a validation cohort. FDG uptake of 17 musculoskeletal sites was evaluated visually and set positive if uptake was at least equal to that of the liver. A decision tree classifier was trained and validated to find the best combination of two positives sites to diagnose PMR. Diagnosis performances were measured first, for each musculoskeletal site, secondly for combination of two positive sites and thirdly using the decision tree created with machine learning.

**Results:** 55 patients with PMR and 85 patients with other inflammatory rheumatisms were included. Musculoskeletal sites, used either individually or in combination of two, were highly imbalanced to diagnose PMR with a high specificity and a low sensitivity. The machine learning algorithm identified an optimal ordered combination of two sites to diagnose PMR. This required a positive interspinous bursa or, if negative, a positive trochanteric bursa. Following the decision tree, sensitivity and specificity to diagnose PMR were respectively 73.2 and 87.5% in the training cohort and 78.6 and 80.1% in the validation cohort.

**Conclusion:** Ordered combination of two visually positive sites leads to PMR diagnosis with an accurate sensitivity and specificity vs. other rheumatisms in a large cohort of patients with inflammatory rheumatisms.

## Keypoints

### Question

Find the best ordered combination of FDG positive musculoskeletal sites with a decision tree classifier to diagnose polymyalgia rheumatica (PMR) vs. other rheumatisms in a large cohort of patients with inflammatory rheumatisms.

### Key Findings

Musculoskeletal sites, used individually or in combination of two, were highly imbalanced to diagnose PMR with a high specificity and a low sensitivity. However, machine learning classifier helped us to build a decision tree to diagnose PMR using two sites. The classifier identified an optimal ordered combination of two sites to diagnose PMR. This required a positive interspinous bursa and, if negative, a positive trochanteric bursa. Following this tree, in a validation cohort, sensitivity and specificity to diagnose PMR were respectively 78.6 and 80.1%.

### Consequences on Patient Care

We proposed an ordered combination of two visually positive musculoskeletal sites to diagnose PMR vs. other inflammatory rheumatisms. It could help clinicians when reporting PET-CT.

## Introduction

[^18^F]Fluorodeoxyglucose positron emission tomography – computed tomography ([^18^F]FDG PET-CT) plays an important role to diagnose polymyalgia rheumatica (PMR) (1) and to rule out other diseases with similar symptoms such as rheumatoid arthritis (RA), relapsing seronegative asymmetric synovitis with pitting oedema, spondylarthritis (SA), or paraneoplastic syndrome (2).

In PMR, [^18^F]FDG can accumulate in various joints, usually shoulders or hips. But, it appeared that uptake in musculoskeletal sites such as ischial bursa, trochanteric bursa and interspinous bursa was more specifically associated with the diagnosis (3, 4). [^18^F]FDG PET-CT used various composite articular scores which proved accurate to diagnose PMR unlike control patients whose sensitivity and specificity ranged from 74 to 90.9% and 79 to 92.4% (5–7) and patients with other rheumatic diseases whose sensitivity and specificity ranged from 85,7 to 92,6% and 85,5 to 90% (3, 4, 8, 9).

Contrary to combinations that are selections of some members of a set regardless of order, permutation of a set is an arrangement of its members into a sequence. Diagnosis values of arrangement of three positive musculoskeletal sites to diagnose PMR were only studied in a cohort of PMR patients vs. control. Results were promising with a sensitivity and specificity above 90% in cohorts of PMR patients and controls (6). However, to the best of our knowledge, no previous study has evaluated the diagnosis values of different permutations of two positive musculoskeletal sites to diagnose PMR patients in a cohort of different rheumatic diseases. Moreover, because it gives an order to assess the different sites, an ordered combination may facilitate PET reporting.

In the present study, the primary aim was to find the best ordered combination of two FDG positive musculoskeletal sites with a decision tree classifier to diagnose polymyalgia rheumatic (PMR) in a large cohort of patients with various inflammatory rheumatisms.

## Methods

### Ethics Approval and Consent to Participate

All procedures performed in studies involving human participants were in accordance with the ethical standards of the institutional research committee and with the 1964 Helsinki Declaration and its later amendments or comparable ethical standards. The study was approved by CECIC Rhône-Alpes-Auvergne, Grenoble, IRB 5921 on 12 November 2019 (IRB number: 5921) and patients provided written informed consent to participate in this study.

### Study Population

In this retrospective study, we reviewed 478 patients' clinical information and [^18^F]FDG PET-CT prescription provided by the Rheumatology and Internal Medicine Departments of our hospital from April 2011 to December 2015.

Inclusion criteria were (1) unclassified diagnosis at the time of PET completion and (2) a delayed final diagnosis of RA following the 2010 American College of Rheumatology/European League Against Rheumatism's criteria (10), of PMR following its 2012 criteria (11) and of SA following the 2009 Assessment of Spondylarthritis International Society's criteria (12). If the rheumatism did not meet these criteria, rheumatologists and internists agreed on a final diagnosis. Yet, some patients remained with a diagnosis of unclassified rheumatism. [^18^F]FDG PET-CT exams were not included in the paraclinical tests used for the final rheumatic diagnosis.

Exclusion criteria were absence of inflammatory rheumatism, namely rheumatic diseases without inflammatory rheumatism (prosthetic loosening, narrowing of the lumber vertebral canal, fracture, fibromyalgia, osteoarthritis, shoulder hand syndrome), infectious disease, inflammatory diseases without musculoskeletal manifestations. Absence of active disease at the time of the [^18^F]FDG PET-CT was also an exclusion criteria. However, patients already treated with corticosteroids or other immunosuppressants treatments were not excluded. When available, the following data was collected: rheumatism activity parameters such as C-reactive protein (CRP), erythrocyte sedimentation rate (ESR), treatment with corticosteroids, or other immunosuppressants (including duration and dose).

### Image Acquisition

Patients fasted for at least 4 h and were injected with an activity of 3–4 MBq/kg of [^18^F]FDG according to current guidelines. Sixty minutes after injection, PET and unenhanced CT images were acquired on a PET-CT scanner: Discovery ST or Discovery 710 Optima 660 (General Electric Healthcare). 85% of the acquisitions extended from the skull to the upper third of the femurs, with the upper extremities situated either along the body or above the head and 15% of the PET/CT involved the entire body. Images reconstruction parameters were identical for PET-CT scanner. A fully 3D time-of-flight iterative reconstruction scheme (VUE Point FX) was used (Ordered subsets expectation maximization algorithm, 24 subsets, 2 iterations) with point spread function modeling (SHARP IR) (13). A low-dose CT scan was acquired for attenuation correction. The full width at half maximum (FWHM) of the gaussian filter was 6.4 mm. The voxel size was 2.7344 × 2.7344 × 3.27 mm^3^. Each voxel in PET images were converted into standard uptake value (SUV) with the following formula: SUV = voxel concentration activity ^*^ patient body weight/decay corrected injected activity (14).

### Image Analysis

[^18^F]FDG uptakes were analyzed at 17 different sites (Supplementary Figures), both articular and peri-articular as proposed by Sondag et al. (5): two shoulders, two acromioclavicular joints (AC joint), and two sternoclavicular joints (SC joint), the most intense interspinous bursa, two hips, two trochanteric bursas (TB), two ischial bursas (IB), two iliopectineal bursas (IPB), and two symphysis pubis enthesis (SPE).

[^18^F]FDG uptake was visually assessed by one experienced nuclear medicine physician with high training in rheumatic disease. Each site was assessed using a standardized 0 to 3 grading system in comparison with the liver uptake (0: no uptake, 1: uptake lower than the liver, 2: moderate uptake, same as that of the liver, 3: higher uptake than the liver) as suggested by the joint procedural recommendation of the European Association of Nuclear Medicine, the Society of Nuclear Medicine and Molecular Imaging and the PET Interest Group (15).

### Input Data for Machine Learning Analysis

We defined a positive site as a site with a score of 2 or 3 (3–5, 15). Bilateral site was considered positive when at least one side was positive. Nine sites were therefore used as input for machine learning analysis: shoulder, AC joint, SC joint, interspinous bursa, hip, TB, IB, IPB, and SPE. So, for each patient, machine learning algorithm was supplied with a vector of 9 numbers composed of 0 or 1 values, following the positivity or not of each site.

### Machine Learning Training and Validation

The machine learning algorithm used to find the best ordered combination of FDG positive musculoskeletal sites was a decision tree classifier. A decision tree is a flowchart-like tree structure in which a root node represents feature, the branch represents a decision rule, and a leaf node represents the outcome. The classifier is an algorithm that partitions the tree in a recursive manner to test which feature of each node - in this case the musculoskeletal site- divides optimally the dataset in two subsets in PMR patients vs. other patients.

The classification method was based on an optimized version of the Classification and Regression Tree (CART) algorithm (16). Heuristic for selecting the splitting criterion is Gini index. It provided a rank to each attribute by explaining the given dataset. Best score attribute was selected as a splitting attribute. The maximum depth of the decision tree was set to two. This machine learning approach was performed using Python (version 3.7) and the open source Scikit-learn package (17).

In order to train and validate decision tree classifier, the study cohort was randomized, stratified on the final diagnosis, on ratio 3: 1 into training and validation cohorts (18). So, classifiers were developed on the training cohort and diagnosis performances were evaluated on the validation cohort.

### Statistical Analysis

Firstly, sensitivity (Se) and specificity (Sp) values for PMR diagnosis vs. other inflammatory rheumatisms were calculated at each site and in combinations of 2 sites (considered positive if both sites were positive).

Secondly, we built a decision tree in order to get the best ordered combination of two sites and measured its Se and Sp values.

Thirdly, we evaluated the diagnostic performance of our algorithm with Se, Sp, positive likelihood ratio (LR+) and negative likelihood ratio (LR–).

Statistical analysis was performed using R software version 3.5.2 (19). Continuous variables were reported as mean (± standard deviation) or median ([range]). Categorical variables were represented as proportions (percentages). All tests were two-sided. Confidence intervals (Cis) were reported at the 95% level, and *p* < 0.05 was considered statistically significant.

## Results

### Patient Characteristics

140 patients with a final diagnosis of inflammatory rheumatisms were selected. Table 1 compares PMR patients' characteristics to patients with other inflammatory rheumatism. No significant difference was found between PMR and other patients as far as age, sex, inflammatory parameters [e.g., C-reactive protein (CRP), erythrocyte sedimentation rate (ESR)] and steroids dose were concerned. Twenty nine (52.7%) patients in the PMR group and 25 (29.4%) among the other inflammatory rheumatisms patients received steroids before [^18^F]FDG PET-CT.

**Table 1:** Patient characteristics from the polymyalgia rheumatica and other inflammatory rheumatisms groups.

**Characteristics**	**PMR patients (*n* = 55)^**#**^**	**Other inflammatory rheumatisms (*n* = 85)**	***p***
Age (median, min-max range), years	70.4 [41–91]	69.0 [34–96]	0.97
Male (*n*, %)	25 (45%)	36 (42%)	0.28
CRP (median, min-max range), (mg/L)	32 [0–270]	17 [0–255]	0.10
ESR (median, min-max range), (mm/h)	41 [3–138]	35.5 [0–165]	0.88
Steroids dose (median, min-max range), (mg/day)	10.5 [2–30] ^*^	8 [4–20]^*^	0.19
Rheumatoid arthritis (*n*, %)	-	42 (30%)	
Spondylarthritis (*n*, %)	-	17 (12.1%)	
Unclassified rheumatism (*n*, %)	-	6 (4,3%)	
Remitting seronegative symmetrical synovitis with pitting oedema (RS3PE) (*n*, %)	-	4 (2,9%)	
Psoriatic rheumatism (*n*, %)	-	4 (2,9%)	
Paraneoplastic rheumatism (*n*, %)	-	4 (2,9%)	
Synovitis-acne-pustulosis-hyperostosis-osteitis (SAPHO) (*n*, %)	-	3 (2.1%)	

*PMR, Polymyalgia rheumatica; CRP, C-reactive protein; ESR, erythrocyte sedimentation rate*.

* *29 patients in the PMR group and 25 in the other inflammatory rheumatisms group*.

# *Among those diagnosed with PMR, 10 patients were also diagnosed with Giant Cell Arteritis (GCA)*.

The cohort was composed of 55 patients with PMR (39,3%), 42 patients with RA (30%), 17 patients with SA (12,1%), 6 patients with unclassified rheumatism (4.3%), 3 patients with SAPHO (2.1%), and an equal number of 4 patients (2,9%) with RS3PE, psoriatic rheumatism and paraneoplastic rheumatism. Among those diagnosed with PMR, 10 patients were also diagnosed with Giant Cell Arteritis (GCA).

The training cohort was composed of 105 patients, the validation cohort was composed of 35 patients and both stratified based on the final diagnosis. No significant difference was found between both cohorts as far as age, sex, inflammatory parameters, and steroids dose are concerned.

### PMR Diagnostic Value of Musculoskeletal Sites Analyzed Individually

Se and Sp values of each musculoskeletal sites were detailed in Table 2. Proportion of positive musculoskeletal site for each group is in Supplementary Table 1. Mean Sp and mean Se of musculoskeletal sites analyzed individually to diagnose PMR were respectively 85 and 49.1%. Symphysis pubis enthesis was the most specific site (94.1 with 95% CI 0.89–0.99) and shoulder was the most sensitive site (71 with 95% CI 0.59–0.83).

**Table 2:** Sensibility, specificity at each musculoskeletal site and at the only combinations of 2 sites with sensitivity above 50% to diagnose patients with polymyalgia rheumatica in the whole cohort of patients with various inflammatory rheumatisms (*n* = 140).

**Musculoskeletal site**	**Sensitivity (95% CI)**	**Specificity (95% CI)**
Shoulder	0.71 (0.59–0.83)	0.65 (0.55–0.75)
Acromioclavicular joint	0.47 (0.34–0.6)	0.82 (0.74–0.9)
Sternoclavicular joint	0.44 (0.3–0.57)	0.86 (0.79–0.93)
Interspinous bursa	0.6 (0.47–0.73)	0.91 (0.84–0.97)
Trochanteric bursa	0.58 (0.45–0.71)	0.93 (0.87–0.98)
Hip	0.42 (0.29–0.55)	0.81 (0.73–0.9)
Ischial bursa	0.6 (0.47–0.73)	0.86 (0.79–0.93)
Symphysis pubis enthesis	0.36 (0.24–0.49)	0.94 (0.89–0.99)
Iliopectineal bursa	0.24 (0.12–0.35)	0.87 (0.8–0.94)
Shoulder + ischial bursaTrochanteric bursa+ ischial bursa	0.51 (0.37–0.63)	0.92 (0.86–0.98)0.95 (0.91–1)
	0.51 (0.37–0.63)	

*CI, Confidence interval*.

### PMR Diagnostic Value of Two Concomitant Positive Musculoskeletal Sites

Combinations of two positive sites were all imbalanced. Indeed, only two combinations had a Se slightly above 50% namely shoulder + ischial bursa with a Se of 51% (95% CI 0.37–0.63) and a Sp of 91.8% (95% CI 0.86–0.98) and trochanteric bursa + ischial bursa with a Se and a Sp of 51% (95% CI 0.37–0.63) and 95.3% (95% CI 0.91–1) (Table 2).

### PMR Diagnostic Value of Machine Learning Analysis

**Figure 1:**
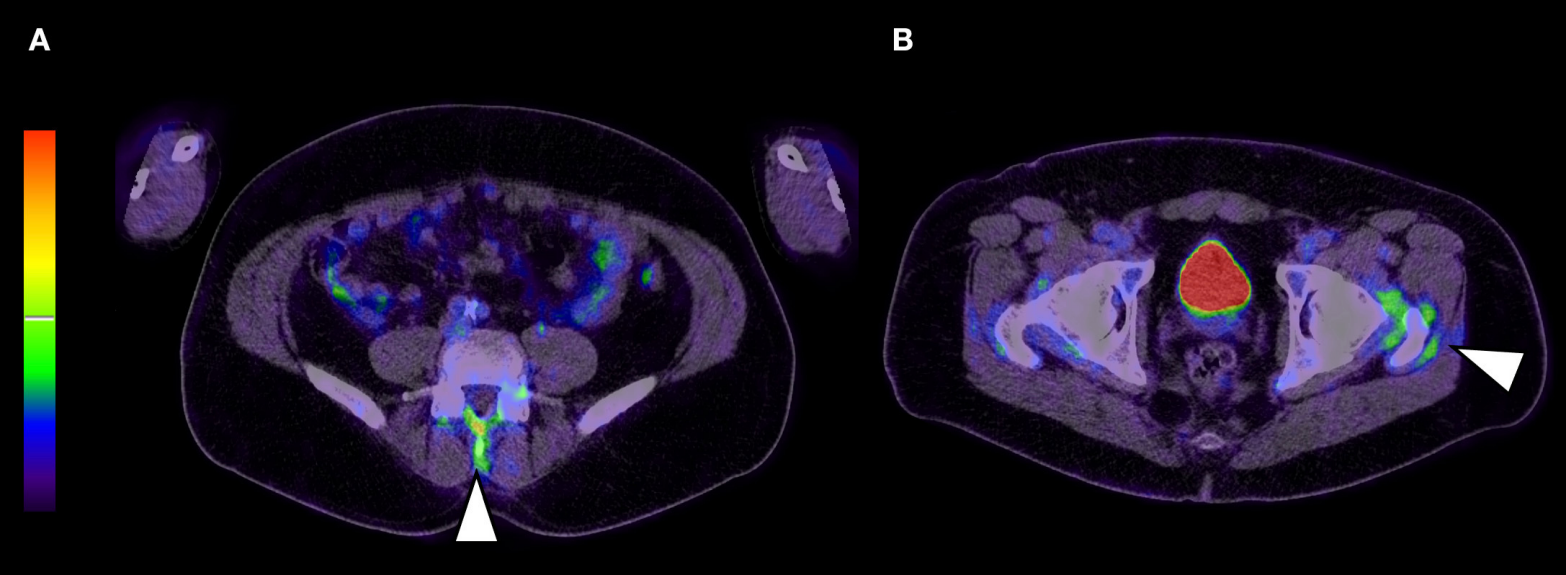
Musculoskeletal sites part of the decision tree are interspinous bursa (**A**; arrow head) and if negative trochanteric bursa (**B**; arrow head).

**Figure 2:**
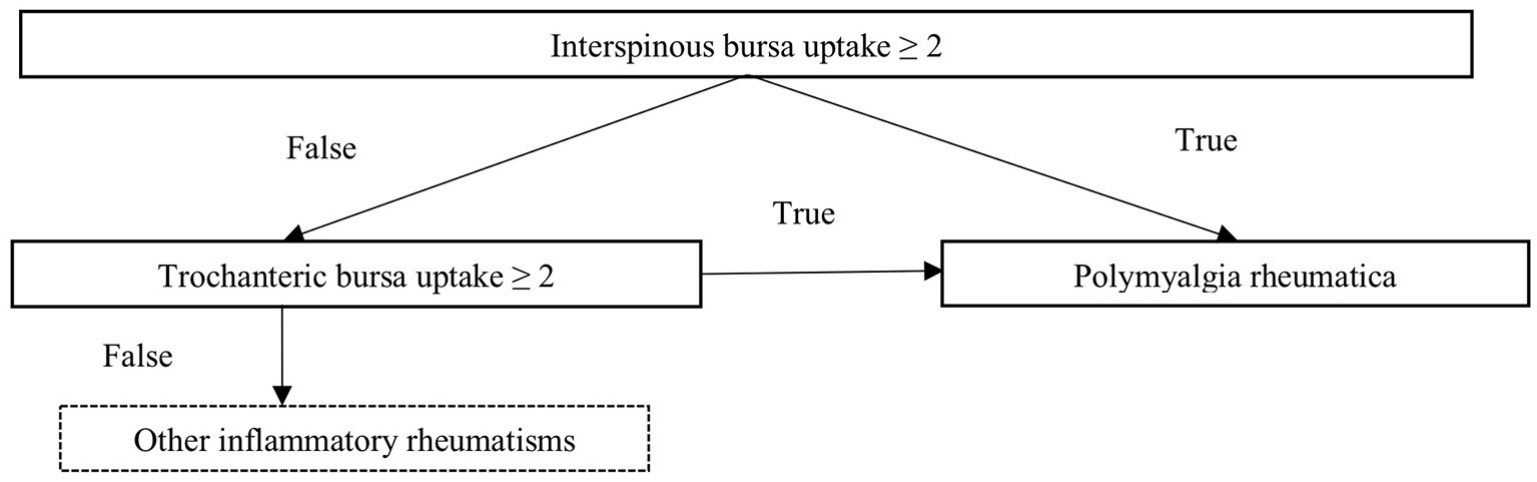
Decision tree to diagnose polymyalgia rheumatica among a group of patients with inflammatory rheumatisms. (2: moderate uptake, same as that of the liver).

According the machine learning classifier output, the optimal way to diagnose PMR was first to evaluate tracer accumulation in interspinous bursa and then, if negative, to evaluate trochanteric bursa tracer uptake. Both musculoskeletal sites are shown on Figure 1 and decision tree is shown on Figure 2. Using this method, Se and Sp to diagnose PMR were respectively 73.2% (95% CI 0.60–0.87) and 87.5% (95% CI 0.77–0.98) in the training cohort and 78.6% (95% CI 0.57–0.1) and 80.1% (95% CI 0.59–1) in the validation cohort. LR+ and LR– were respectively 5.85 (95% CI 2.98–11.49) and 0.31 (95% CI 0.18–0.51) in the training cohort and 3.95 (95% CI 1.6–9.72) and 0.27 (95% CI 0.1–0.75) in the validation cohort. Pooled results are summarized in Supplementary Table 2.

## Discussion

We used machine learning to define a short decision tree able to detect PMR patients among a large retrospective cohort of patients with inflammatory rheumatisms. Machine learning enabled to enhance the diagnostic value of musculoskeletal site assessed visually. Indeed, used individually or in combination of two concomitant positive sites, sensitivity and specificity were highly imbalanced and inappropriate to diagnose PMR. On the other hand, machine learning defined order of two sites allowed accurate specificity and sensitivity to diagnose PMR. We purposely used a decision tree classifier as a white box model, a system in which the inner logic is intelligible thus, results were easily explained and interpreted. It eased clinical translation of machine learning approaches to the clinics (20). Indeed, assessment of the musculoskeletal sites should be prioritized in clinical routine thanks to the created decision tree.

Splitting rules of the decision tree were based on a positive interspinous bursa or, if negative, a positive trochanteric bursa. The selected sites in decision tree were already explored in the context of patients diagnosed with PMR. Firstly, interspinous [^18^F]FDG uptake was correlated with MRI to interspinous bursitis (21). It is also described as a very informative site to diagnose PMR, with a specificity ranging from 82.4 to 100% (3, 7). Its pooled LR+ 4 (95% CI 1.84–8.71) for diagnosis of PMR is the highest among all musculoskeletal sites according to a recent review (22). Therefore, our study results are in accordance with the literature. Secondly, [^18^F]FDG uptake at trochanteric bursa was due to the trochanteric bursitis. It was one of the most consistent findings in PMR so its inclusion in decision tree is in accordance with the literature (3, 23). Although easy to apply, these splitting rules gave visual score-based decision tree a good accuracy. Its LR+ and LR– are consistent with those of pooled composite [^18^F]FDG-PET/CT scores which were respectively 3.91 (95% CI 2.42–6.32) and 0.19 (95% CI 0.10–0.36) in a recent systemic review about the diagnostic value of [^18^F]FDG-PET/CT in PMR (22).

In the literature, global performance of [^18^F]FDG PET for PMR diagnostic whatever criteria used, ranges from 74 to 92.4% for Se and from 79 to 92.6% for Sp (3–9). This is in line with our results, in a large cohort and even after validation in an independent cohort. Studies proposed various composite articular scores in two different conditions: either vs. control patients or vs. patients with other rheumatic disease. For example, one approach was to define the total skeletal score, which reflected uptake in the 12 studied articular regions. Sensitivity and specificity were respectively measured at 85.1 and 87.5%, controls being non-PMR-rheumatic or inflammatory disease (7). Another approach was to look for a minimum number of positive sites - at least 3 out of 17- in order to be more effective in clinical routine. Sensitivity and specificity to diagnose PMR patients vs. control group were respectively of 74 and 79% (5). Regarding patients with other rheumatic or inflammatory disease, sensitivity, and specificity were respectively 86 and 85,5% (9). However, in both studies, the diagnostic value of each site individually was not taken into account. Recent studies encouraged to focus on positivity of musculoskeletal sites more specifically associated with the diagnosis of PMR. One study identified ischial tuberosities, peri articular shoulder and interspinous bursa as 3 specific sites allowing a PMR diagnosis. It suggested positivity of these 3 sites resulted in a high sensitivity of 90.9% and a high specificity of 92.4% vs. control patients (5, 6). However, it was unusual to find the peri-articular shoulder site among the three, as its specificity was usually low in various studies (3, 7). Furthermore, diagnostic accuracy of more specific musculoskeletal sites was evaluated in a small cohort of patients with various rheumatic diseases. It suggested positivity of 2 sites among 3 assessed (ischial tuberosity, greater trochanter, and lumbar spinous process) resulted in a high sensitivity (85.7%) and specificity (88.2%) (3). It was close to the ordered combination of musculoskeletal sites established by machine learning to assess PMR diagnosis in our large cohort. Splitting rules were based on a positive interspinous bursa or, if negative, a positive trochanteric bursa.

There were some limitations to our study, the first being its retrospective design and descriptive nature. Inclusion criteria were heterogeneous as various rheumatic diseases were considered. Moreover, whole body examination was not always performed as some patients were not referred for rheumatic pathology and [^18^F]FDG PET-CT were not always performed at the same time as the disease evolved, both at initial evaluation or during follow up. Besides, 29/55 (53%) of PMR patients had already received glucocorticoids when [^18^F]FDG PET-CT was performed. Glucocorticoids may have decreased sensitivity of [^18^F]FDG PET-CT with reduced incidence of abnormal finding and FDG uptake intensity (5) however, our diagnostic accuracy remains reliable. In addition, although decision tree suggests preferential articular or peri-articular sites to analyse to differentiate PMR in a large cohort of patients with inflammatory rheumatisms, assessment of PET should not be limited to 2 sites: full examination of all sites must be done. Lastly, we did not include any quantitative analysis in this machine learning approach because the objective was to propose a robust and reproductive clinic visual method known to be less sensitive to acquisition conditions than quantification methods (24). Finally, our findings have to be validated in multicentric prospective studies with larger cohorts. Methodological improvement would be to develop an automatic segmentation of each musculoskeletal site with automatic ratio quantification to liver uptake.

## Conclusion

We proposed an ordered combination of two visually positive musculoskeletal sites to diagnose PMR thanks to machine learning. Splitting rules were based on a positive interspinous bursa or, if negative, a positive trochanteric bursa. It was validated in a large cohort of patients with inflammatory rheumatisms and was able to diagnose patient with an accurate sensitivity and specificity. It could help clinicians with PET-CT reporting.

## Data Availability Statement

The original contributions presented in the study are included in the article/Supplementary Material, further inquiries can be directed to the corresponding author.

## Ethics Statement

The studies involving human participants were reviewed and approved by CECIC Rhône Alpes Auvergne, Grenoble, IRB 5921. The patients/participants provided their written informed consent to participate in this study.

## Author Contributions

JA, MC, and AF especially contributed to acquire data. MA, MS, LO, SM, and LD performed the clinical evaluations, treatments, and follow-up evaluations of the patients. FC, JA, and AF contributed to conception and design. CB, BB, and CV to revised the manuscript and approved the final content of the manuscript. AF contributed to interpret data. NP, CM, AK, and FC to enhanced the intellectual content. All authors read and approved the final manuscript.

## Conflict of Interest

The authors declare that the research was conducted in the absence of any commercial or financial relationships that could be construed as a potential conflict of interest.
