# Supplementary material for: Decision Tree With Only Two Musculoskeletal Sites to Diagnose Polymyalgia Rheumatica Using [18F]FDG PET-CT
Source: Front Med (Lausanne). 2021 Feb 17;8:646974. doi: 10.3389/fmed.2021.646974 (PMC7928279; doi:10.3389/fmed.2021.646974)
Supplement: Supplementary file 1 [file Data_Sheet_1.docx]

**Supplementary Figures**

[^18^F]FDG uptakes were analysed at seventeen different sites both articular and peri-articular. Supplementary figures 1 to 9 showed fused ^18^[F]FDG PET-CT images for each musculoskeletal site.


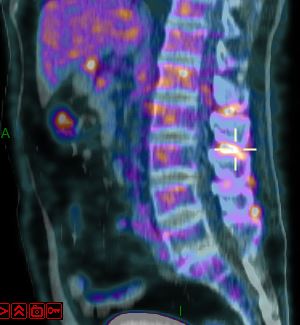


**Supplementary figure 1**: FDG uptake at the interspinous bursa


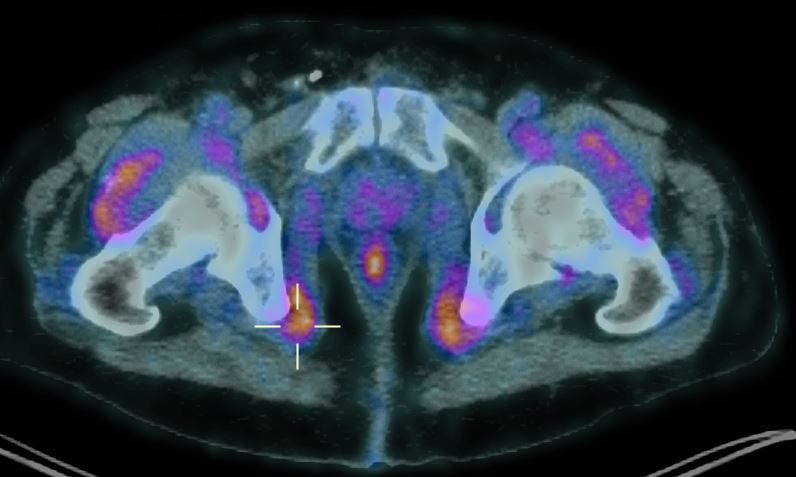


**Supplementary figure 2**: FDG uptake at the ischial bursa


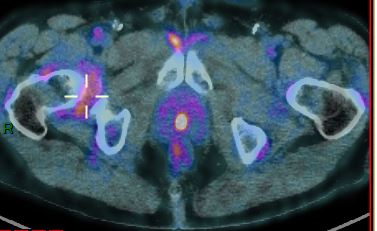


**Supplementary figure 3**: FDG uptake at the right hip


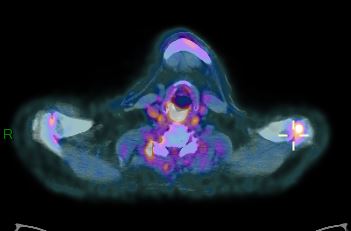


**Supplementary figure 4**: FDG uptake at the acromioclavicular site


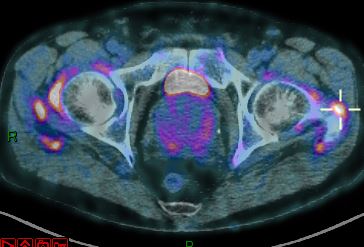


**Supplementary figure 5**: FDG uptake at the trochanteric bursa


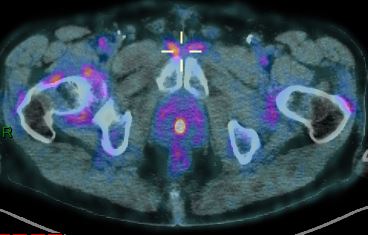


**Supplementary figure 6**: FDG uptake at the symphysis pubis enthesis


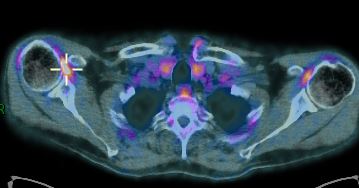


**Supplementary figure 7**: FDG uptake at the shoulder


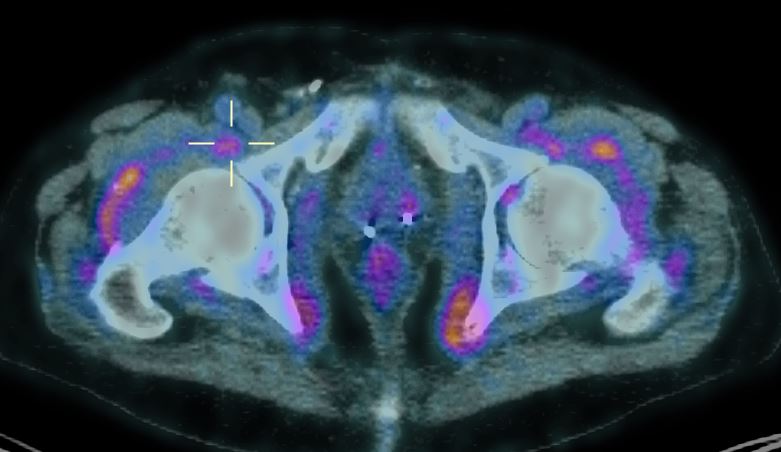


**Supplementary figure 8**: FDG uptake at Iliopectineal bursa


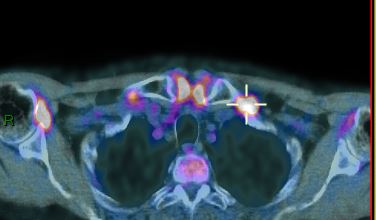


**Supplementary figure 9**: FDG uptake at sternoclavicular site
