# Supplementary material for: Decision Tree With Only Two Musculoskeletal Sites to Diagnose Polymyalgia Rheumatica Using [18F]FDG PET-CT
Source: Front Med (Lausanne). 2021 Feb 17;8:646974. doi: 10.3389/fmed.2021.646974 (PMC7928279; doi:10.3389/fmed.2021.646974)
Supplement: Supplementary file 2 [file Data_Sheet_2.docx]

**Supplementary tables**

| **Musculoskeletal sites** | **Polymyalgia rheumatica patients (n=55)**  Number of positive sites | **Others inflammatory rheumatisms patients**  **(n=85)**  Number of positive sites | ***p*** |
| --- | --- | --- | --- |
| Shoulder | 39 (71%) | 30 (35%) | < 0.001 |
| AC joint | 26 (47%) | 15 (18%) | < 0.001 |
| SC joint | 24 (44%) | 12 (14%) | < 0.001 |
| Interspinous bursa | 33 (60%) | 8 (9%) | < 0.001 |
| TB | 32 (58%) | 6 (7%) | < 0.001 |
| Hip | 16 (29%) | 7 (8%) | < 0.001 |
| IB | 23 (42%) | 6 (7%) | < 0.001 |
| SPE | 14 (25%) | 2 (2%) | < 0.001 |
| IPB | 9 (16%) | 5 (6%) | 0.158 |

**Supplementary table 1:** Incidence of abnormal findings in qualitative analysis (^18^F-FDG uptake score ≥ 2) at each musculoskeletal site.

| **Musculoskeletal sites (n=140)** | **Sensitivity** | **Specificity** | **PPV** | **NPV** | **Accuracy** |
| --- | --- | --- | --- | --- | --- |
| Shoulder | 0,71 | 0,65 | 0,57 | 0,77 | 0,67 |
| Acromioclavicular joint | 0,47 | 0,82 | 0,63 | 0,71 | 0,69 |
| Sternoclavicular joint | 0,44 | 0,86 | 0,67 | 0,70 | 0,69 |
| Trochanteric bursa | 0,58 | 0,93 | 0,84 | 0,77 | 0,79 |
| Hip | 0,42 | 0,81 | 0,59 | 0,68 | 0,66 |
| Ischial bursa | 0,6 | 0,86 | 0,73 | 0,77 | 0,76 |
| Symphysis pubis enthesis | 0,36 | 0,94 | 0,80 | 0,70 | 0,71 |
| Interspinous bursa | 0,6 | 0,91 | 0,80 | 0,78 | 0,79 |
| Iliopectineal bursa | 0,24 | 0,87 | 0,54 | 0,64 | 0,62 |
| **Combination of 2 musculoskeletal sites (n=140)** |  |  |  |  |  |
| Shoulder + ischial bursa | 0,51 | 0,92 | 0,80 | 0,74 | 0,76 |
| Trochanteric bursa + ischial bursa | 0,51 | 0,95 | 0,88 | 0,75 | 0,78 |
| Interspinous bursa + shoulder | 0,49 | 0,94 | 0,84 | 0,74 | 0,76 |
| Shoulder + acromioclavicular joint | 0,47 | 0,86 | 0,68 | 0,72 | 0,71 |
| Shoulder + trochanteric bursa | 0,47 | 0,94 | 0,84 | 0,73 | 0,76 |
| Interspinous bursa + ischial bursa | 0,45 | 0,96 | 0,89 | 0,73 | 0,76 |
| Interspinous bursa + trochanteric bursa | 0,44 | 0,98 | 0,92 | 0,73 | 0,76 |
| Acromioclavicular joint + ischial bursa | 0,42 | 0,93 | 0,79 | 0,71 | 0,73 |
| Shoulder + sternoclavicular joint | 0,40 | 0,92 | 0,76 | 0,70 | 0,71 |
| interspinousbursa + acromioclavicular joint | 0,38 | 0,95 | 0,84 | 0,70 | 0,73 |
| interspinous bursa + sternoclavicular joint | 0,38 | 0,95 | 0,84 | 0,70 | 0,73 |
| Shoulder + hip | 0,36 | 0,89 | 0,69 | 0,68 | 0,69 |
| Acromioclavicular joint + trochanteric bursa | 0,36 | 0,96 | 0,87 | 0,70 | 0,73 |
| Hip+ ischial bursa | 0,35 | 0,95 | 0,83 | 0,69 | 0,71 |
| Acromioclavicular joint + sternoclavicular joint | 0,33 | 0,94 | 0,78 | 0,68 | 0,70 |
| Sternoclavicular joint + ischial bursa | 0,33 | 0,95 | 0,82 | 0,69 | 0,71 |
| Interspinous bursa + hip | 0,31 | 0,96 | 0,85 | 0,68 | 0,71 |
| Interspinous bursa + symphysis pubis enthesis | 0,31 | 0,96 | 0,85 | 0,68 | 0,71 |
| Sternoclavicular joint + trochanteric bursa | 0,31 | 0,96 | 0,85 | 0,68 | 0,71 |
| Trochanteric bursa + hip | 0,31 | 0,96 | 0,85 | 0,68 | 0,71 |
| Shoulder + symphysis pubis enthesis | 0,29 | 0,98 | 0,89 | 0,68 | 0,71 |
| Acromioclavicular joint + hip | 0,29 | 0,94 | 0,76 | 0,67 | 0,69 |
| Trochanteric bursa + symphysis pubis enthesis | 0,27 | 0,98 | 0,88 | 0,67 | 0,70 |
| Ischial bursa + symphysis pubis enthesis | 0,27 | 0,96 | 0,83 | 0,67 | 0,69 |
| Sternoclavicular joint or + hip | 0,24 | 0,93 | 0,68 | 0,65 | 0,66 |
| Sternoclavicular joint + symphysis pubis enthesis | 0,24 | 0,95 | 0,76 | 0,66 | 0,67 |
| Acromioclavicular joint + symphysis pubis enthesis | 0,22 | 0,96 | 0,80 | 0,66 | 0,67 |
| Shoulder + iliopectineal bursa | 0,20 | 0,92 | 0,61 | 0,64 | 0,64 |
| Hip + iliopectineal bursa | 0,20 | 0,89 | 0,55 | 0,63 | 0,62 |
| Acromioclavicular joint + iliopectineal bursa | 0,18 | 0,92 | 0,59 | 0,63 | 0,63 |
| Trochanteric bursa + iliopectineal bursa | 0,18 | 0,95 | 0,71 | 0,64 | 0,65 |
| Hip + symphysis pubis enthesis | 0,18 | 0,95 | 0,71 | 0,64 | 0,65 |
| Ischial bursa + iliopectineal bursa | 0,18 | 0,94 | 0,67 | 0,64 | 0,64 |
| Interspinous bursa + iliopectineal bursa | 0,16 | 0,96 | 0,75 | 0,64 | 0,65 |
| Sternoclavicular joint + iliopectineal bursa | 0,15 | 0,92 | 0,53 | 0,62 | 0,61 |
| Symphysis pubis enthesis + iliopectineal bursa | 0,15 | 0,95 | 0,67 | 0,63 | 0,64 |
| **Following decision tree algorithm** |  |  |  |  |  |
| Training cohort (n=105) | 0,73 | 0,88 | 0,73 | 0,85 | 0,80 |
| Validation cohort (n=35) | 0,79 | 0,80 | 0,79 | 0,84 | 0,82 |

**Supplementary table 2**: sensibility, specificity, PPV, NPV and accuracy at each musculoskeletal site, at each combination of 2 musculoskeletal sites and following our decision tree algorithm to diagnose patients with polymyalgia rheumatica in the whole cohort of patients with various inflammatory rheumatisms.
